# Expectations from a Home Cooking Program: Qualitative Analyses of Perceptions from Participants in “Action” and “Contemplation” Stages of Change, before Entering a Bi-Center Randomized Controlled Trial

**DOI:** 10.3390/nu15092082

**Published:** 2023-04-26

**Authors:** Rani Polak, Adi Finkelstein, Maggi A. Budd, Brianna E. Gray, Hanni Robinson, Julie K. Silver, Mark D. Faries, Amir Tirosh

**Affiliations:** 1 Spaulding Rehabilitation Hospital, Department of Physical Medicine and Rehabilitation, Harvard Medical School, Boston, MA 02129, USA; 2 Center of Lifestyle Medicine, Sheba Medical Center, Sackler School of Medicine, Tel-Aviv University, Tel-Hashomer 52621, Israel; 3 Department of Nursing, Faculty of Life and Health Sciences, Jerusalem College of Technology, Jerusalem 95483, Israel; afinkels@g.jct.ac.il; 4 Department of Spinal Cord Medicine, VA Boston Healthcare System, Department of Psychiatry, Harvard Medical School, Boston, MA 02130, USA; margaret.budd@va.gov; 5 Translational and Clinical Research Centers, Massachusetts General Hospital, Boston, MA 02114, USA; bgray4@mgh.harvard.edu; 6 Medical School, University of Nicosia, Nicosia 2408, Cyprus; rubinsohn.c@live.unic.ac.cy; 7 Massachusetts General Hospital, Brigham & Weman’s Hospital, Spaulding Rehabilitation Hospital, Department of Physical Medicine and Rehabilitation, Harvard Medical School, Boston, MA 02129, USA; julie_silver@hms.harvard.edu; 8 Texas A&M School of Public Health and College of Medicine, Bryan, TX 77807, USA; fariesmd@gmail.com; 9 Division of Endocrinology, Diabetes and Metabolism, Sheba Medical Center, Sackler School of Medicine, Tel-Aviv University, Tel-Hashomer 5262161, Israel; amir.tirosh@sheba.health.gov.il

**Keywords:** home cooking, stages of change, personalized medicine, lifestyle medicine

## Abstract

Home cooking is an emerging strategy to improve nutrition; however, the literature lacks reports about patient expectations from culinary interventions. Personalized medicine utilizes knowledge about a person’s genes; yet, behavioral factors, such as participant “readiness” to make a change, may also impact treatment preferences and outcomes. The purpose is to explore the expectations of participants in different stages of change from a home cooking intervention. Participants were recruited to a randomized controlled trial evaluating the impact of a home cooking intervention on weight. Stage of change assessed by a validated University of Rhode Island Change Assessment scale and expectations through an open-ended questionnaire. Sixteen (21%) participants were in the action stage of change, and 59 (79%) were in the contemplation stage. Participants from both groups shared similar expectations to achieve healthy eating and lifestyle goals and to adopt sustainable change. However, action group expectations also included expanding existing culinary knowledge and change of habits; the contemplation group expectations also included acquiring culinary knowledge, improving self-regulatory skills, and obtaining guidance and support. While action group participants were looking to expand existing knowledge and techniques, contemplation group participants were focusing on acquiring culinary knowledge and skills. This can potentially contribute to developing effective, personalized nutrition interventions.

## 1. Introduction

Adherence to a healthy diet is associated with a decreased risk of non-communicable diseases, such as cardiovascular disease, type 2 diabetes, and cancer [1,2], as well as improved immunity [3], cognitive function [4,5], and longevity [6,7]. However, adherence to a healthy diet pattern is challenging in a real-world setting. Barriers include availability and cost of healthy foods [8], stress [9], time for shopping and food preparation [10], and knowledge of cooking skills [10]. Home cooking has been suggested as a strategy to improve adherence to a healthy diet [11,12,13]. Although culinary medicine programs (i.e., health-related home cooking education) are emerging, there is currently no consensus about the learning objectives and curricular domains of these programs [14].

While patient-centered care utilizes individual-specific health needs and preferences to influence healthcare interventions [15], the literature lacks reports about expectations from home cooking interventions. In addition, only a few studies have looked at participants’ expectations of nutrition interventions [16]. For example, participants from an internet-based workplace nutrition intervention conveyed a need for a tailored approach to set specific goals and social support to facilitate adherence and noted other desirable program features [16]. These include recipes, interactivity, nutritional information, shopping tips, cost-saving information, and a companion smartphone app [16]. Personalized medicine improves outcomes through knowledge about a person’s genes that improve diagnostics and drug preferences [17]. However, other personalization aspects, such as personality and behavioral factors, may also impact treatment preferences and outcomes, especially when tailoring a lifestyle modification intervention.

Adopting healthy behaviors is not a static event; rather, behavioral change is a nonlinear, dynamic process. The transtheoretical model serves as a useful heuristic stage model for understanding a person’s motivation or “readiness” to make a change and describes the cognitive change processes that promote movement through the stages [18]. This theory, which became one of the most popular and enduring in the field of health promotion, describes five distinct stages that align with a person’s readiness to change. This includes precontemplation (not yet considering a behavioral change), contemplation (thinking about it), preparation (intending to act), action (made changes but for less than six months), and maintenance (changes have sustained 6 + months). A number of studies suggested that using stage-based dietary interventions is effective in achieving positive nutritional goals and behaviors, such as increasing vegetable and reducing fat consumption [19].

The central concept within the transtheoretical model is that individuals are more likely to experience success in changing [nutrition] behavior when they engage in strategies and cognitive processes appropriate to their readiness to make the change [19]. Thus, appropriate strategies and behavioral change processes can be implemented once a person’s stage is determined [20]. Cognitive processes prevalent in the contemplation stages are consciousness-raising, self-reevaluation, dramatic relief, environmental reevaluation, and social liberation [21]. Similarly, cognitive processes of change for individuals in action can be counterconditioning, stimulus control, reinforcement management, helping relationships, and self-liberation. Matching the cognitive processes of change with the stage of change can constructively inform interventions and increase the chance of success [20,21].

Since home cooking may be an effective strategy to improve adherence to a healthy diet [11,12,13], and there is a dearth of information regarding participant expectations from home cooking interventions, this study aims to (1) explore participant goals when enrolling in a home cooking-based nutrition intervention, and (2) examine whether participants in different stages of change (i.e., contemplation or action stage) have different needs and expectations.

## 2. Materials and Methods

### 2.1. Setting and Participants

This is a bi-center randomized controlled trial aimed at evaluating the impact of a remote home cooking intervention on the nutrition and weight of participants who are overweight or obese (this study was approved by both sites’ institutional review boards; protocol #2018P002115; NCT03823469). Participants were recruited at Spaulding Rehabilitation Hospital (Spaulding), Boston, Massachusetts, United States, and Sheba Medical Center (Sheba), Tel Aviv, Israel. Inclusion criteria included body mass index (BMI) equal to or greater than 27.5 kg/m^2^ and equal to or lower than 35 kg/m^2^; primary food provider of the household who consumes fewer than five home-cooked lunches and dinners per week; and age 25–70 years. Participants were randomly assigned to either an intervention or control group. Both groups completed two 30-min nutritional counseling sessions. The intervention group also completed a 3-month culinary coaching telemedicine program (i.e., twelve 30-min culinary coaching sessions) [22,23] (CCTP), while the control group was provided with access to nutritional education resources (e.g., brochures, websites).

### 2.2. Data Collection

The study self-administrated questionnaires that were collected at baseline (before randomization) included: (1) Stages of change in relation to home cooking as assessed by the validated University of Rhode Island Change Assessment Scale (URICA) Readiness Score (a 32-item self-administered Likert scale questionnaire) [24], which was modified using a factor analysis [25] to specifically assesses participants’ stages of change in regard to home cooking; and (2) open-ended questionnaire asking participants to respond to share their goals, expectations, concerns, and challenges.

### 2.3. Data Analysis

Participants’ stage of change in relation to home cooking habits before the beginning of the program was analyzed (i.e., one cohort including both the intervention and control group participants). Then, participants were categorized for qualitative analysis into one of two groups: contemplation (URICA scale < 11) and action (URICA scale > 11) (URICA = 11 were classified manually through experts opinion). Participants in the precontemplation and maintenance groups were not observed.

Conventional content analysis [26] was performed on each group separately. This analysis is usually appropriate when the program (i.e., CCTP) is innovative, existing theory or research literature on a phenomenon is limited, and data collection is done for the first time. Qualitative data were analyzed manually [27]. Analysis began with reading and re-reading the data responses of each group separately. The next step was open coding [28] (i.e., breaking the data into discrete parts and labeling them), then codes were sorted and gathered into categories and subcategories. To enhance the trustworthiness and credibility of the data, we used triangulation (i.e., the use of multiple methodologies, resources, or practices to minimize researcher bias) [29,30,31,32]. Codes were created inductively by the group qualitative researcher (AF) and were reviewed by the principal investigator (RP). Sorting and gathering the codes into categories and subcategories was followed by debriefing correspondences with the team behavioral health expert (MF) and neuropsychologist (MB). For thoroughness, explanations and quotes to support our analysis are provided [33], and the manuscript follows the COREQ checklist for qualitative studies [34]. Demographics were analyzed using a t-test for quantitative (continuous) variables and chi-squared for categorical data.

## 3. Results

All 75 study participants (39 from the Sheba site and 36 from the Spaulding site) completed the questionnaires. Of these, 16 (21%) were in the action stage of change regarding home cooking habits, and 59 (79%) were in the contemplation stage of change. Full demographic details are presented in Table 1, separated by action and contemplation groups.

Both active and contemplated groups were similar in relation to gender, age, ethnic background, employment status, household income, and the highest level of education. Table 2 includes the categories of participant expectations and concerns, summarized according to their stage of change.

### 3.1. Contemplation Stage

#### 3.1.1. Participant Goals and Expectations

Acquire culinary knowledge and skills—Participants in the contemplation stage wanted to acquire knowledge about healthy cooking: “ [to] learn how to cook healthier and more nutritious food, and slowly give all the members of the household more proper eating habits” (SMC34); “My expectations are to learn healthy cooking tips that are both practical and understandable for people who are not good cooks” (SP04); “and learn cooking and baking ways that will be nutritious and varied enough to improve my nutritional lifestyle” (SMC11); “I joined the program to learn to cook properly, so I can lower my high sugar and cholesterol levels” (SMC12).

Participants explained they wanted to improve their cooking techniques, which may improve their culinary capacities, for example: “enhance my abilities on cooking” (SP24); “More familiarity with meal prep will reduce time and frustration” (SP18); “to know how to manage the cooking at home” (SMC29).

Improve self-regulatory skills—Participants in the contemplation stage desired to learn how to implement new culinary knowledge and skills through improved self-regulation, such as prioritizing, planning, organizing, and time management: “My goals would be to prioritize home cooking more often and expect that I will be able to improve this frequency” (SP07); “I hope I will have a better decision-making process [related to home cooking]” (SP29); planning and organizing: “I love to cook and love healthy food but find it difficult to plan and find the time to cook healthy meals at home. I am hopeful that this program will help me feel more confident in doing this” (SP22); “prep meals ahead of consumption” (SP18); and time management skills: “knowing how to incorporate healthy eating/cooking when life gets busy, and there is not much time” (SP04).

Adopt a sustainable change—Participants in the contemplation stage expected their knowledge, skills, and abilities to translate to sustainable, lasting changes in their and their family’s routine and cooking habits: “I know I really want to change my and my children’s eating habits” (SMC21); “I also do not eat enough during the day for meals, and so I snack all night. I would like more routine” (SP11); “Goals—stability and cooking consistent meals (especially dinners)” (SP30B); “expect a substantial change in-home cooking habit” (SMC38).

Achieve healthy eating and lifestyle goals—Participants in the contemplation phase wished to apply their understanding of healthy eating and food choices in order to lose weight and improve their health: “I am looking to understand nutrition balance and portion control” (SP18); “raise awareness for a healthy diet” (SMC28); “Hoping to lose weight and to do so in a healthy way” (SP11); “To improve health indicators and also to lose weight” (SMC29); “learn to eat healthy cooked food instead of junk food, and work to promote my health” (SMC25); “My hope is that this program will bring my focus back to nutrition” (SP26). A few participants mentioned exercise together with healthy eating and a healthy lifestyle: “I am interested in joining the program so I can try to change the components of my diet to a healthier diet while incorporating proper exercise” (SMC14).

Get guidance and support—Participants in the contemplation stage looked for a framework so they could make a change: “I have made a change however I feel I need to be in some framework that will help me with habits and perseverance” (SMC21); “I decided to try and join something more controlled with the option of more careful support and follow-up” (SMC5). They asked for guidance and supervision from experts: “I have been trying to make healthier lifestyle choices over the last 2 years related to diet and exercise. I think participating in this program and being able to learn tools from experts would help my efforts” (SP07); “ [I expect] guidance for a healthier lifestyle. Close supervision and assistance with weight loss” (SM35); “I love to cook. I think that with your guidance I can get on the ‘king’s road’ and change my poor dietary behavior” (SMC16). Participants also mentioned their expectations for support and encouragement: “Also, having support to help me identify and overcome barriers to cooking” (SP04); “I need someone to accompany me and encourage me to succeed” (SMC9); “I know how to cook but the implementation of actually doing it is challenging. I need someone to steer me in the right direction” (SP17).

#### 3.1.2. Participants Concerns

Pre-existing culinary challenges—Participants in the contemplation stage mentioned that several factors they expected to improve upon, such as poor habits and lack of experience and practice in cooking, were also barriers that might make it difficult for them during the program: “I barely cook from scratch; in that way, I think I am getting stuck to an unhealthy lifestyle. I am interested to change this habit” (SP15); “It will be challenging to cook more” (SMC9); “I hardly cook at home for reasons of laziness and boredom from the food after once or twice and I would like to improve on that” (SMC4). Participants also mentioned their fear of not meeting the program requirements and that the fear of failure will stand as a barrier to their success: “I am afraid that I will not be able to cook everything required in the workshop” (SMC36); “Persistence in the menus of an external factor seems to be a very serious challenge” (SMC26).

Insufficient self-regulation abilities—Participants in the contemplation stage expressed a number of specific self-regulation challenges in adopting home cooking. Examples include: (1) time management: “accepting the positive benefits it [healthy home cooking] can have if I take the time and effort to do it” (SP29); (2) control: “My challenges are how to not eat as much and portion control” (SP05); “not giving into eating temptations” (SP07); “The challenges [are] not to be tempted to eat junk food” (SMC31); and (3) self-organizing; “I love to cook and love healthy food but find it difficult to plan” (SP22). As noted above from participant expectations, these self-regulatory challenges are also expectations for improvement, thus providing an opportunity for the program to help develop and implement these specific abilities and skills.

Implementation in daily routine—Participants in the contemplation stage noted challenges with assimilating home and healthy cooking in their daily life. Emotional challenges were also acknowledged, such as finding motivation: “It will be challenging to cook more” (SMC09); “challenges would be finding Motivation to stick with this program” (SP22)”; “greatest challenge is to find the motivation to cook for myself” (SP28). Other specific challenges were (1) costs: “Worries about the costs of healthy foods” (SP04); “The costs of cooking (for one) are a concern” (SP11); (2) time: “I love to cook and love healthy food but find it difficult to plan and find the time to cook healthy meals at home” (SP22); “I work 3–4 evening shifts per week, and I am a single mom. Time is limited in the schedule” (SP17); and (3) lack of family support: “My challenge will be to make time to prepare meals for myself when I know that at least at the beginning, the rest of the household will not be full partners in the dietary change” (SMC34). Participants also mentioned socializing as a challenge: “ We socialize a lot and it is difficult for me to make healthy choices in restaurants or parties”. (SP31).

Overcoming obstacles for sustainability—Despite goals for sustainability in behavior change, participants in the contemplation stage expressed concerns about their ability to persist and maintain their future diet and healthy eating achievements in view of their (negative) past experience: “It has been difficult to stay on track with every diet I have tried on my own. I am hoping that with guidance I can do it this time” (SP25); “I really want to lose weight and how surprising—I have a hard time. Maybe with your help, I will make a difference in my life” (SMC19); being out with friends and making wise eating choices” (SP11); “I am concerned on being successful but without a long-lasting positive impact” (SP24); “My concerns are that I won’t be able to keep up to new good habits” (SP04); “The challenges [are] not to be tempted to eat junk food. To maintain a controlled daily healthy day routine” (SMC31). “the main challenge is perseverance especially after the program ends. Therefore, it is good that this is a long program, and you can get used to it and it will be easy to continue” (SMC22).

### 3.2. Action Stage

#### 3.2.1. Participant Goals and Expectations

Expanding culinary knowledge and techniques—Participants in the action stage wished to improve culinary techniques: “I would like to learn how to cook for just 1–2 people” (SP06); “expanding my knowledge of cooking methods” (SMC10) and specifically healthy cooking techniques: “To learn to prepare healthy meals from scratch (SP08)”; “I would like to develop good cooking” (SP10); “I am joining to learn more about healthy cooking” (SP02); “Home cooking: learn how to eat/prepare foods better” (SP34). Participants mentioned improving self-regulation characteristics that will help them improve their abilities in cooking, such as motivation “to cook well for myself” (SP02); and efficiency of not having to “spend hours in the kitchen cooking healthy food” (SMC37).

Adopting sustainable change: It was important to participants in the action stage to have knowledge and tools for a long-term change in their culinary behavior and in their lifestyle that will last permanently: “dramatic and sustainable change to my approach to eating” (SP01); “I would like to find a sustainable way to meal plan/execute” (SP01); “I joined the program to get help to lose weight. Most importantly is to get tools to maintain it” (SMC15); “to succeed big time and make a turn in my life. Food will not manage me. I will handle it using the knowledge I will learn in the program. In this way, I will embark on a new path” (SMC15); “I would like to learn strategies to change my lifestyle more permanently” (SP23).

Participants in the action stage mentioned fun and enjoyment as part of the process: “I want meal planning, shopping and cooking to be fun and enjoyable” (SP01); “I would love to do this enthusiastically thanks to the knowledge and tools I acquire. I believe I will have fun in the kitchen” (SMC37).

Achieve healthy eating and lifestyle goals—Participants in the action stage wished to be healthier eaters: “I want to be a healthier eater (SP06)”; “I would like to improve at eating healthy” (SP08); “Tired of diets, just want to eat healthier” (SP13); “I want to improve my diet and become more self-sufficient. I rely too much on eating out where I cannot control what I eat. I have special medical considerations, so this is important” (SP21). Participants in the action stage wished to improve and enhance their knowledge about healthy food and healthy diet: “gain knowledge of healthy foods” (SP02); “enhance knowledge of healthy eating/cooking from experts” (SP03); “Get better educated to make changes on a healthy diet which could improve overall health” (SP08); “help me make an order [in health eating] and provide me with the information I lack to eat healthily during my intense day” (SMC37); “I would like to learn to appreciate cooking and eating healthfully as well” (SP10).

Change of habits: Participants in the action stage wished to learn about healthy habits that will enable them to promote a more healthful life: “Joining the program to enhance knowledge of healthy eating/cooking from experts. I expect to learn healthy habits” (SP03); “I would like to develop good cooking and eating habits that will promote a more healthful life” (SP10).

Participants emphasized their wish for an overall change in their lifestyle: “A change in my lifestyle (balancing work and taking care of myself and my family, making time to eat during work hours, eating not in front of a TV)” (SMC10); “to learn about using the new technologies for personal change and health reasons” (SP02); “I am hoping I can learn how to use my time wisely and to learn how to cook” (SP06).

Participants wished to keep/lose weight but consider this as part of an overall behavior change in their habits and lifestyle: “All my life I have struggled with weight. I want to make a lifestyle change to live right” (SMC23); “Would like to lose weight from cooking more often and eating more nutritious meals, better portion control” (SP01); “I would like to eat properly and maintain the correct weight for my size and to become physically fit” (SP12); “to change lifestyle with prepping and eating healthy foods to lose weight and reduce inflammation” (SP13).

#### 3.2.2. Participants’ Concerns in Achieving Their Goals

The main challenges participants in the action stage considered were continuity and sustainability of healthy changes: “No concerns. Challenge will be being consistent” (SP03); “Only challenges would be to continue what I learned” (SP08); “Other concern is not get sabotaged by friends and family when they are not eating healthy things” (SP13); “I am worried that maybe the new eating routine will not fit into my daily routine. I hope so” (SMC37); ”We socialize a lot and it is difficult for me to make healthy choices in restaurants or parties” (SP31).

## 4. Discussion

In this study, we analyzed the expectations and concerns of participants enrolled in a nutrition intervention focused on home cooking. Both contemplated and active participants shared similar expectations to achieve healthy eating and lifestyle goals and to adopt sustainable change. However, while participants in the active stage of change were looking to expand existing knowledge and techniques, participants in the contemplation stage of change were focusing on acquiring culinary knowledge and skills, including self-regulatory skills. In addition, while the concerns of participants in the action group were broadly related to the continuity and sustainability of healthy changes, the concerns of the contemplative participants were more explicit. Concerns included preexisting culinary challenges, insufficient self-regulation abilities, and implementation into the daily routine.

Current home cooking education is focused on culinary skills [14]. The categories derived from this qualitative study can create a strong foundation of patient-centered pillars for designing a personalized home cooking program for participants in the early to middle stages of change. As such, the needed culinary knowledge and skills should be taught (i.e., acquiring culinary knowledge and skills) aligned with the common goals of health for participants and their families (i.e., achieving healthy eating and lifestyle goals) while also helping participants connect behavior to more internalized reasons and motivations, such as enjoyment, pleasure, taste, and personally held values (i.e., promote autonomy/self-determination, self-empowering through stages of change). Yet, an understanding of how to implement home cooking in participants’ own lives is needed. This requires a set of behavior change strategies and tips to help participants self-regulate their behavior and manage challenges in their home and social environments, especially with consideration to perseverance over barriers and recovery from relapse for sustainable change and routine formation. This will require ongoing support, access to tips and further knowledge and skills.

There were notable differences in the hierarchy of needs required to promote changes between participants in the contemplation and action stages, which is congruent with cognitive processes of change described in the literature [21]. Coinciding with lower-level needs, contemplation stage participants expressed a need for foundational skills both in culinary and self-regulation skills and shared more concerns and anticipated challenges than action participants. Cognitive processes prevalent in the contemplation stages are consciousness-raising, self-reevaluation, dramatic relief, environmental reevaluation, and social liberation [21]. Contemplated participant responses in this cohort validated this description. Specifically, participants freely stated they need enhanced confidence and learning skills that are personalized to their needs and values (i.e., consciousness-raising, dramatic relief). As with others with ambiguity to make a behavioral change [35], contemplation individuals were aware of both the pros and cons of adopting home cooking (i.e., decisional balance). For example, indulging in food/drink has many rewards and reinforcements (e.g., feels and tastes good, social aspects) as well as cons (e.g., weight gain and harm to health). Appropriate for the contemplation stage, these individuals have ambivalence and concerns about lasting change.

Participants in the action stage related to home cooking were mostly looking to enhance and expand the confidence and culinary skills that they already possessed, and they also stated fewer concerns and challenges. Participants’ responses in this cohort were aligned with the known cognitive processes of action participants [21], emphasizing behavioral processes of stimulus control (e.g., removing triggers to unwanted behavior and adding cues for the desired behavior) and contingency management (increasing the rewards for positive behavior and decreasing the reward for the unwanted behavior). Like others in an action stage of change, participants in this home cooking study have modified their thinking and some behavior (s) toward desired change but may lack particular skills or solid confidence or consistency to be in the next stage. The main concern for those in the action stage was sustainability in an already good situation. For example, they frequently used “more” and “better” which indicates the concept was already present but needed enhancement.

For decades now, studies have demonstrated compelling evidence for tailoring interventions according to a person’s stage of readiness for behavioral change related to improved nutritional benefits [18,20,36,37]. Extending this study data and integrating cognitive processes can also inform home cooking programs. Knowing when individuals are in a particular stage of change related to home cooking, interventions can be customized to specific needs, preferences, and barriers. Patient-centered home cooking interventions for contemplative participants may include support/guidance, new skills, and addressing concerns about failing. Personalized interventions for action participants may focus on improving the current level of confidence, honing culinary and related skills, and enhancing internal motivations. Action participants expressed a higher level of expectations (e.g., becoming “better” at an already acquired skill) and appreciation (e.g., appreciating the need for a permanent shift).

Strengths of this study include a binational cohort and the categorization of responders according to an individual’s home cooking-related stages of change. Limitations include a small number of participants in the action stage of change and the lack of participant social diversity. Further studies are needed to explore participant goals and expectations from home cooking interventions depending on stages of change and whether these goals and expectations vary according to sociodemographic status and ethnic background. Further research is also needed to determine whether a personalized intervention, based on behavioral factors such as stages of change, will result in positive, sustainable home cooking habits.

## 5. Conclusions

This study adds to the literature as there is little information about expectations from nutrition interventions, categorized by participant stages of change. This can potentially contribute to the development of personalized, effective nutrition interventions. In summary, the findings from this study are promising for home cooking programs seeking a genuinely individualized approach.

## Figures and Tables

**Table 1:** Demographic of participants in the culinary coaching study, divided into action and contemplation stages of change.

	Action (*n* = 16)	Contemplation (*n* = 59)	Total (*n* = 75)	*p*-Value
Mean age, years (SD)	49.63 (14.58)	46.08 (12.87)	46.84 (13.29)	0.35
Female	10 (62%)	40 (68%)	50 (67%)	0.69
Marital Status				0.34
Married	5 (31%)	29 (49%)	34 (45%)	
Living together	3 (19%)	4 (7%)	7 (9%)	
Never married	6 (38%)	15 (25%)	21 (28%)	
Divorced	1 (6%)	9 (15%)	10 (13%)	
Widow	1 (6%)	2 (3%)	3 (4%)	
US ethnic background				0.12
American Indian	0 (0%)	2 (8%)	2 (6%)	
Asian	0 (0%)	4 (17%)	4 (11%)	
Black American African	2 (17%)	0 (0%)	2 (6%)	
White	9 (75%)	17 (71%)	26 (72%)	
Hispanic	1 (8%)	1 (4%)	2 (6%)	
Israel ethnic background				
Jewish	4 (100%)	34 (97%)	38 (97%)	
Arab (Christian)	0 (0%)	1 (3%)	1 (3%)	
Employment status				0.66
Employed	14 (88%)	51 (86%)	65 (87%)	
Retired	2 (12%)	4 (7%)	6 (8%)	
Student	0 (0%)	1 (2%)	1 (1%)	
Other	0 (0%)	3 (5%)	3 (4%)	
Yearly household income				0.28
Below the average	0 (0%)	7 (12%)	7 (10%)	
Around the average	5 (33%)	12 (21%)	17 (24%)	
Above the average	10 (67%)	37 (66%)	47 (66%)	
Highest level of education				0.15
High School degree	2 (12%)	9 (15%)	11 (15%)	
Bachelor’s degree or higher	12 (75%)	49 (83%)	61 (81%)	
Other	2 (12%)	1 (2%)	3 (4%)	

**Table 2:** Categories of participant expectations and concerns.

**Contemplation Stage Participants**	
**Goals and Expectations**	**Concerns**
Acquire culinary knowledge and skills	Pre-existing culinary challenges
Improve self-regulatory skills	Insufficient self-regulation abilities
Adopt sustainable change *	Implementation in daily routine
Achieve healthy eating and lifestyle goals *	Overcoming obstacles for sustainability
Get guidance and support	
**Action Stage Participants**	
**Goals and Expectations**	**Concerns**
Expanding culinary knowledge and techniques	Continuity and sustainability of healthy changes
Adopt sustainable change *	
Achieve healthy eating and lifestyle goals *	
Change of habits	

Note: * indicates similarities between groups.

## Data Availability

The datasets used and/or analyzed during the current study are available from the corresponding author upon reasonable request.
